# Volume-regulated anion channels conduct ATP in undifferentiated mammary cells and promote tumorigenesis in xenograft nude mouse

**DOI:** 10.3389/fcell.2024.1519642

**Published:** 2025-01-15

**Authors:** Kishio Furuya, Hiroaki Hirata, Takeshi Kobayashi, Hiroshi Ishiguro, Masahiro Sokabe

**Affiliations:** ^1^ Department Human Nutrition, Nagoya University Graduate School of Medicine, Nagoya, Japan; ^2^ Mechanobiology Laboratory, Nagoya University Graduate School of Medicine, Nagoya, Japan; ^3^ Human Information Systems Labs, Kanazawa Institute of Technology, Hakusan-shi, Ishikawa, Japan; ^4^ Department Physiology, Nagoya University Graduate School of Medicine, Nagoya, Japan

**Keywords:** ATP release, mammary epithelial cells, breast cancer, hypo-osmotic, sphingosine-1-phosphate, LRRC8A, VRAC, xenograft

## Abstract

The high interstitial ATP concentration in the cancer microenvironment is a major source of adenosine, which acts as a strong immune suppressor. However, the source of ATP release has not been elucidated. We measured ATP release during hypotonic stress using a real-time ATP luminescence imaging system in breast cell lines and in primary cultured mammary cells. In breast cell lines, ATP was released with a slowly rising diffuse pattern, whereas in primary cultured cells, ATP was intermittently released with transient-sharp peaks. The diffuse ATP release pattern changed to a transient-sharp pattern by cholera toxin treatment and the reverse change was induced by transforming growth factor (TGF) β treatment. DCPIB, an inhibitor of volume-regulated anion channels (VRACs), suppressed the diffuse pattern. The inflammatory mediator sphingosine-1-phosphate (S1P) induced a diffuse ATP release pattern isovolumetrically. Knockdown of the A isoform of leucine-rich repeat-containing protein 8 (LRRC8A), the essential molecular entity of VRACs, using shRNA suppressed the diffuse pattern. In the nude mouse xenograft model, LRRC8A knockdown suppressed the tumorigenesis of subcutaneously implanted breast cancer cells. These results suggest that abundantly expressed VRACs are a conduit of ATP release in undifferentiated cells, including cancer cells.

## 1 Introduction

Extracellular ATP is a ubiquitous mediator of local intercellular signaling within the body (Burnstock and Di Virgilio, 2013). Extracellular ATP is quickly hydrolyzed by ecto-ATPases and is maintained at a concentration near zero in the interstitial fluids of unstressed tissues. However, the ATP concentration is considerably high at sites of inflammation or in cancerous tissues (Pellegatti et al., 2008; Di Virgilio, 2012) despite the abundance of ecto-ATPase. The functions of extracellular ATP in cancer are gradually being clarified (Alvarez et al., 2022; Vultaggio-Poma et al., 2020). One major characteristic of cancer is the suppression of the immune attack on tumor cells in the cancer microenvironment. This makes cancer immune therapy difficult. A chronically increased level of adenosine was shown to contribute to this immunosuppression via multiple pathways, including the inhibition of T cells and dendritic cells, and the activation of regulatory T cells (Antonioli et al., 2013a). A major source of adenosine is the hydrolysis of ATP by the ecto-ATPases, CD39 and CD73. CD39 is expressed in regulatory T cells (Antonioli et al., 2013b), and both enzymes exist abundantly in the cancer microenvironment. These enzymes are now considered to be therapeutic targets (Häusler et al., 2014). The adenosine pathway to suppress antitumor immune responses also affected the efficiency of immunotherapy in a recent clinical trial using anti-PD-1/PD-L1 monoclonal antibody (mAbs) as immune checkpoint inhibitors (Beavis et al., 2015b). Furthermore, adenosine receptor 2A blockade on T cells significantly enhanced the efficacy of anti-PD-1 mAb and increased the survival of mice inoculated with CD73^+^ tumors (Beavis et al., 2015a).

Despite increasing evidence to support the importance of adenosine pathways in the cancer microenvironment, the source of ATP, which is itself a major source of adenosine, remains unclear. The existence of high concentrations of ATP at sites of inflammation or in the cancer microenvironment is readily accepted due to the presence of destroyed or dying cells. However, these cells do not release much ATP and/or the release is not sustained. To maintain a high ATP concentration in the cancer microenvironment, ATP must be continuously released in a regulated manner; however, the mechanisms through which this occurs are not known. In the present study, we revealed a diffuse prolonged pattern of ATP release following hypotonic stress in breast cell lines, which was blocked solely by DCPIB, a blocker of volume-regulated anion channels (VRACs) (Decher et al., 2001; Friard et al., 2017), suggesting the involvement of these channels in the diffuse release of ATP.

VRACs are a major mechanism of vertebrate cell volume regulation and also participate in numerous physiological and pathophysiological processes, including cancer, edema, cell proliferation, migration, angiogenesis and apoptosis (Okada et al., 2009; Pedersen et al., 2016). The cell volume dramatically changes with the cell cycle, and cell cycle progression is a cell-size dependent process (Ginzberg et al., 2015). VRACs are differentially regulated throughout the cell cycle, and the inhibition of VRACs suppresses proliferation in various types of cells, including hepatocytes, endothelial cells, smooth muscle cells and cancer cells (Pedersen et al., 2016). In 2014, leucine-rich repeat containing 8 family A (LRRC8A) was identified as an indispensable component of VRACs (Voss et al., 2014; Qiu et al., 2014), although at least one other family member is needed to mediate the VRAC current. LRRC8 is a distantly pannexin-1-related protein family and forms a hetero hexamer (Jentsch et al., 2016; Abascal and Zardoya, 2012), which may be the reason for the extremely varied properties of VRACs. We herein confirmed the contribution of VRACs to the release of ATP by the knockdown of LRRC8A using shRNA.

VRACs are activated by hypotonic stress. In higher organisms, systemic osmolality is maintained by multilevel homeostatic control. Nevertheless, dramatic changes in osmolality occur in the kidney and gastrointestinal tract with the induction of the influx of large amounts of saccharides, amino acids, and sodium ions. Furthermore, cells experience frequent fluctuations in volume due to unbalanced transmembrane fluxes of ions and nutrients or macromolecule synthesis and degradation. Under pathological conditions in the brain, ischemia, hyponatremia, and epilepsy cause astrocytic edema (cell swelling) (Mongin, 2016). In patients with cancer, the degradation of protein synthesis, including albumin, causes cachexia and edema (Fazzari and Singh, 2016). VRACs are also activated by various chemical stimuli, including sphingosine-1-phosphate (S1P). S1P is a signaling lysophospholipid and an inflammatory mediator like bacterial lipopolysaccharides, which abundantly exists in the cancer microenvironment. We herein report that prolonged diffuse ATP release via VRACs was induced by hypotonic stress and S1P application in undifferentiated breast cell lines and enhanced by treatment with transforming growth factor (TGF) β, a carcinogenic agent, and reduced by treatment with cholera toxin, an anti-carcinogenic agent. We also investigated whether this ATP release affects carcinogenesis *in vivo* using a nude mouse xenograft model. An earlier version of this paper appeared in pre-print style (Furuya et al., 2021b).

## 2 Materials and methods

### 2.1 Cell culture

Cell lines: The cancerous breast cell lines MCF7 (AKR-211, MCF-7/GFP, Cell Biolabs, Inc., San Diego, CA, United States) and MDA-MB231 (AKR-201, MDA-MB231/GFP, Cell Biolabs, Inc.) were cultivated in DMEM/F12 (Wako Pure Chemical, Osaka, Japan) supplemented with 10% FBS; the non-carcinogenic breast epithelial cell line MCF10A (CRL-10317, ATCC, Manassas, VA, United States) was cultivated on a collagen-coated dish in HuMEC (Gibco, Thermo Fisher Scientific, Waltham, MA, United States); at 37°C under 5% CO_2_.

Primary culture: Mammary glands were dissected from lactating ICR mice (Japan SLC, Hamamatsu, Japan) after lethal deep anesthesia (pentobarbital Na, a combination anesthetic with medetomidine, midazolam and butorphanol, or halothane). Mammary epithelial cells were isolated and cultured, as described previously (Nakano et al., 1997) using Dispase II (Godo Shusei Co., Tokyo, Japan) and collagenase (Type III; Worthington, Freehold, NJ, United States).

For the measurements, cells of the primary culture and several cell lines were cultured on collagen gel (Cellmatrix type I-A, Nitta Gelatin, Osaka, Japan) on a 14- or 22-mmφ cover glass (#1, Matsunami Glass Ind. Ltd. Osaka, Japan) for 1–4 days at 37°C under 5% CO_2_, in sub-confluent to confluent conditions. In some experiments, 3 types of cells (e.g., different cell lines or different shRNA treated cells) each on 3 separated collagen-gel patches were cultured simultaneously on a 22-mmφ cover glass, which made it easy to compare the responses under the same conditions of cultivation and measurement. To identify the cell types on a cover glass under a microscope, a marker was drawn along one edge using an oily marking pen. Control cells were placed near the marker, while shA1 and shA2 cells were positioned on the right and left sides of the control cells, respectively. In some cases, the cells were cultured on collagen sheet. Cholera toxin (100 ng/mL) (Wako), cholera toxin B subunit (Wako) or TGFβ (10 ng/mL) (Wako) was added to the culture medium for 3 h to 3 days before measurement in some cases.

### 2.2 Experimental setup

The cells on the cover glass were set in a small perfusion chamber (approximately 100 µL in volume) on the stage of an upright microscope (BX51WI; Olympus, Tokyo, Japan) with a 1× (Plan UW, NA0.04; Nikon, Tokyo, Japan), 4× (340 Fluor XL, NA0.28; Olympus) or 10× (Plan Apo, NA0.45; Nikon) objective lens. The medium was replaced with DME/F12 buffered with 10 mM HEPES (pH 7.4) (Gibco) containing 40%–50% luciferin-luciferase solution (see below). Medium changes (300 μL) were performed via capillary action for approximately 30 s without any mechanical effect of flow. Hypotonic solutions were made by adding a solution with 1.05 mM CaCl_2_ + 0.7 mM MgCl_2_ (30%–50% [v/v]), which keeps the Ca^2+^ and Mg^2+^ concentration constant in hypotonic solutions. The osmolality of each medium was DME/F12-HEPES: 303 mosm; 30% hypotonic solution: 218 mosm; 50%hypotonic solution: 155 mosm. In some cases, just distilled water was used to make hypotonic solutions, but no marked difference on the ATP response was observed. DCPIB (4-[(2-Butyl-6,7-dichloro-2 -cyclopentyl-2,3-dihydro-1 -oxo-1H-inden-5-yl) oxy] butanoic acid, TOCRIS, Bristol, UK), sphingosine-1-phosphate (Huzzah S1P, Human Serum Albumin/sphingosine-1-phosphate Complex; Avanti Polar Lipids, Inc., Alabaster, AL, United States) and other active reagents were added to the perfusion medium.

### 2.3 Real-time imaging of the ATP release

The ATP release was measured in real-time using a luminescence imaging system that has been previously described (Furuya et al., 2014). Luciferin-luciferase ATP bioluminescence was detected using a high-sensitivity camera system simultaneously with infrared-DIC imaging to monitor the cells. An osmolality adjusted luciferin-luciferase solution (Lucifer HS Set: Kikkoman Biochemifa Co., Tokyo, Japan; or Luciferase FM plus: Bioenex Inc., Hiroshima, Japan) was added to the perfusate with 40%–50% volume. After standing for 15 min after a medium change, ATP-dependent luminescence was detected with a high-sensitivity EMCCD camera (Cascade 512F; Photometrics, Tucson, AZ, United States) equipped with a cooled image intensifier (C8600-04; Hamamatsu Photonics, Hamamatsu, Japan). Images were acquired at a frequency of 2 Hz with an exposure time of 500 ms using the MetaMorph software program (ver. 7.8; Molecular Devices, San Jose, CA, United States) in stream acquisition mode. For the data analyses, image smoothing was usually conducted by calculating the average of six sequential images. In our system, DCPIB (100 µM) itself does not affect luciferin-luciferase bioluminescence by ATP. ATP imaging experiments were performed at 30°C ± 2°C.

### 2.4 Measurements of regulatory volume decrease (RVD)

It is known that VRACs are responsible for the regulatory volume decrease (RVD) that occurs after a volume increase by hypotonic stress. Volume changes during hypotonic stress (30%) were monitored using perpendicular cross-sectional image of cells that constitutively expressed GFP in the cytosol. This was accomplished with a confocal X-Z-T scan (LSM510, Carl Zeiss, Oberkochen, Germany) utilizing a 63 × NA 1.4 objective, a scan speed of 4 s and a 10 s interval. The cell volume is inversely proportional to the fluorescence intensity of a specific unit area of the cytosol. We measured the fluorescence intensity of designated area of the cross-sectional image where no intracellular organelles were observed. The change in cell volume was calculated as the reciprocal of the fluorescence intensity and normalized to a value obtained just prior to stimulation.

### 2.5 Real-time polymerase chain reaction (qPCR)

The expression of LRRC8 family members (A to E) was measured by reverse transcription (RT) qPCR. mRNA specimens were isolated from the cells grown on collagen-gel in 24-well dishes using a NucleoSpin RNAplus RNA isolation kit (Macherey-Nagel, Dueren, Germany) and converted to cDNA with SuperScript IV VILO Master Mix (Invitrogen, Thermo Fisher Scientific, Waltham, MA, United States) or SuperScript III First-Strand Synthesis System (Invitrogen, Thermo Fisher Scientific). The expression was determined by RT-qPCR using a LightCycler 480 or Nano (Roche, Mannheim, Germany) with SYBR Green I Master Mix (Roche) and quantitative primers (Perfect Real Time Primer, Takara, Shiga, Japan, Supplementary Table S1). The expression was normalized to GAPDH within each sample.

### 2.6 LRRC8A knockdown with shRNA

LRRC8A silencing with shRNA was performed using retrovirus mediated gene transfer as described previously (Hirata et al., 2017). To generate retrovirus expressing shRNA against LRRC8A, the target sequences (Supplementary Table S1) were inserted into the pSUPER.retro.puro retroviral vector (OligoEngine, Seattle, WA, United States). For control, a non-targeting sequence 5′-ATA​GTC​ACA​GAC​ATT​AGG​T-3′ was introduced. The shRNA-containing vector was co-transfected with the pE-ampho vector into HEK293T cells using GeneJuice transfection reagent (Merck Millipore, Burlington, MA, United States). Supernatants containing viral particles were collected 48 h after the transfection, filtered through 0.45-μm syringe filters, and used for infection into three breast cell lines in the presence of 8 μg/mL Polybrene (Sigma-Aldrich, St. Louis, MO). Infected cells were selected with 1.5 μg/mL puromycin (Sigma-Aldrich).

### 2.7 Nude mouse xenograft experiment

The animal experiment protocol was approved by the Animal Experiment Review Committee, Graduate School of Medicine, Nagoya University (approval no. M230399-001). Four-week-old female nude mice, BALB/cSlc-nu/nu, were purchased from Japan SLC (Hamamatsu, Japan) and maintained 12-h dark/light cycle with pathogen-free conditions in the Division of Experimental Animals, Nagoya University. MDA-MB231 cells, both LRRC8A knockdown (shA1) and non-targeting control, were cultivated 3–4 days until confluent in 60 mmφ dishes and harvested to make a cell suspension of 1 × 10^7^ cells/mL. 100 μL of LRRC8A knock down and non-targeting control cells were injected subcutaneously into the left and right sides of the lower back of each nude mouse, respectively. The growth of the xenograft tumors was monitored for 4–5 weeks. Tumor size was measured using a digital caliper in two dimensions (L: length and W: width), and tumor volume was estimated as an ellipsoid using equation L*W^2^*4*pai/3/8. Tumor growth was also monitored using an *in vivo* imaging system (IVIS Spectrum, PerkinElmer Co., Ltd., Shelton, CT, United States), with fluorescent image of GFP expressed in MDA-MB231 cells.

## 3 Results

### 3.1 Hypo-osmotic stress induced two types of ATP release depending on the cell conditions

**FIGURE 1:**
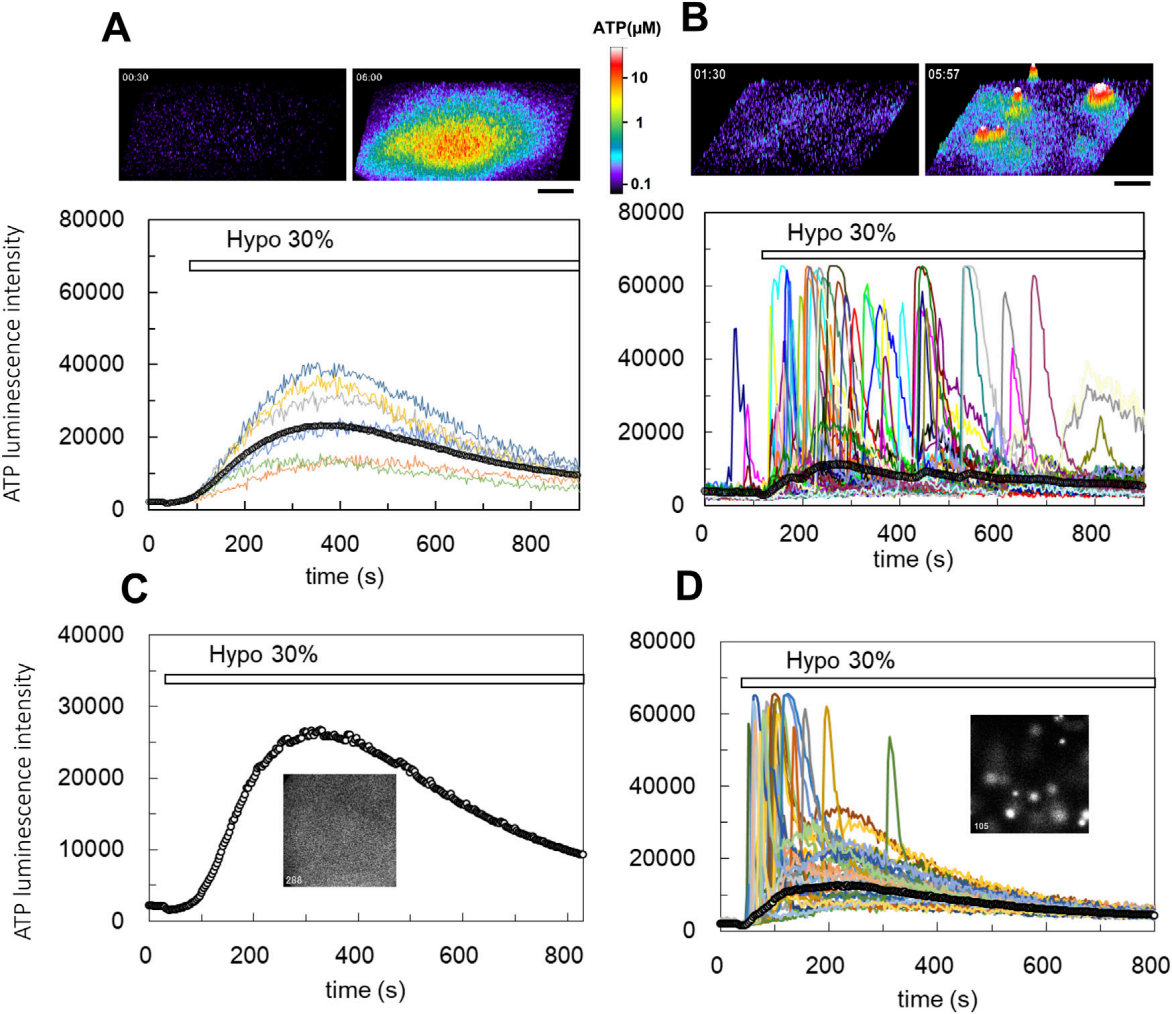
Real-time ATP luminescence imaging reveals different kinetics and patterns of ATP release induced by hypotonic stress in breast cell lines **(A)** and primary cultured mammary epithelial cells **(B)**. **(A)** Upper panels; hypotonic stress (30%, 70% osmolality, 218 mosm) induced diffuse ATP release in a breast cell line (MDA-MB231). The responses were slow-rising and had a diffuse appearance (“diffuse” response). ATP release is shown in the 3D intensity profile (see also Supplementary Movie S1). Lower trace; the time course of the luminescence change observed at several points of cell culture and the time course of the average luminescence intensity over the entire observed area (a line with open circles). Other breast cell lines (MCE7, MCF10A) exhibited similar diffuse responses following hypotonic stress. **(B)** Upper figures; hypotonic stress (30%) induced transient ATP release events in primary mammary epithelial cells from a lactating mouse. Sharp responses occurred intermittently at different time points in several randomly distributed cells (“transient-sharp” response). ATP release is shown in the 3D intensity profile (see also Supplementary Movie S2). Lower traces; the time course of luminescence changes in each responding cell and the average luminescence intensity in the entire observed area (a line with open circles). **(C, D)** The pattern of ATP release was changed from “diffuse” to “transient-sharp” after treatment with the cholera toxin. Hypotonic stress (30%) induced slow-rising and diffuse ATP release in MCF10A cells (C, see also Supplementary Movie S3). Cholera toxin (100 ng/mL) treatment for 7 h changed the pattern of ATP release to the transient-sharp (D, see also Supplementary Movie S4). The cells used in C and D were sister cultures obtained from a single plating. All cholera toxin-treated (3 h–3 days) experiments including other cell lines (MCF7, MDA-MB231) (over 23) showed similar results. Scale bar indicates 500 μm.

Hypotonic (hypo-osmotic) stress was applied to induce ATP release from several types of mammary epithelial cells, and distinct differences between primary cultured cells and breast cell lines were revealed for the first time using our ATP imaging system. In a breast cell line (MDA-MB231), 30% hypotonic stimulation (70% osmolality, 218 mosm) induced a slow-rising ATP release response with a diffuse appearance (Figure 1A; Supplementary Movie S1; hereafter referred to as the “diffuse” pattern). In this release pattern, we could not identify individual ATP-releasing cells. In contrast, in primary cultures of mammary epithelial cells from lactating mice, 30% hypotonic stimulation induced the transient release of ATP, which occurred intermittently in several randomly distributed cells in the colony (Figure 1B; Supplementary Movie S2; hereafter referred to as the “transient-sharp” pattern). The duration of each peak was several dozen seconds.

The other breast cell lines, MCF7 carcinoma and MCF10A non-carcinogenic, and a human lung carcinoma cell line (A549) (Furuya et al., 2014) exhibited a diffuse ATP release pattern. Conversely, other primary cultured subepithelial fibroblasts from the rat small intestine (Furuya and Furuya, 2007) exhibited a transient-sharp ATP release pattern (Supplementary Figure S1).

The two types of ATP responses are interchangeable. The diffuse ATP responses observed in MCF10A breast cell line (Figure 1C; Supplementary Movie S3) and other carcinogenic cell lines (MCF7 and MDA-MB231) were modified to a transient-sharp pattern (Figure 1D; Supplementary Movie S4) following cholera toxin treatment (100 ng/mL) for 7 h (range: 3 h to 3 days). Interestingly, in the transient-sharp pattern, the local peak concentration of released ATP during each event was significantly higher than that in the diffuse pattern, however, the average ATP concentration in the entire area was greater and longer in the diffuse pattern (Figures 1C,D; lines with open circles).

The two types of ATP responses were not exclusive. Even in cholera toxin-untreated cells, transient-sharp ATP release occasionally occurred in a few cells immediately after hypotonic stress overlapped with the diffuse pattern. In addition, spontaneous ATP release with a transient-sharp pattern was occasionally observed in the absence of hypotonic stress. The appearance of the transient-sharp pattern appeared to depend on the conditions of each cell in culture; however, the mechanisms through which this pattern is induced remain to be elucidated.

### 3.2 DCPIB blocks the diffuse release of ATP, but not the transient-sharp release of ATP

**FIGURE 2:**
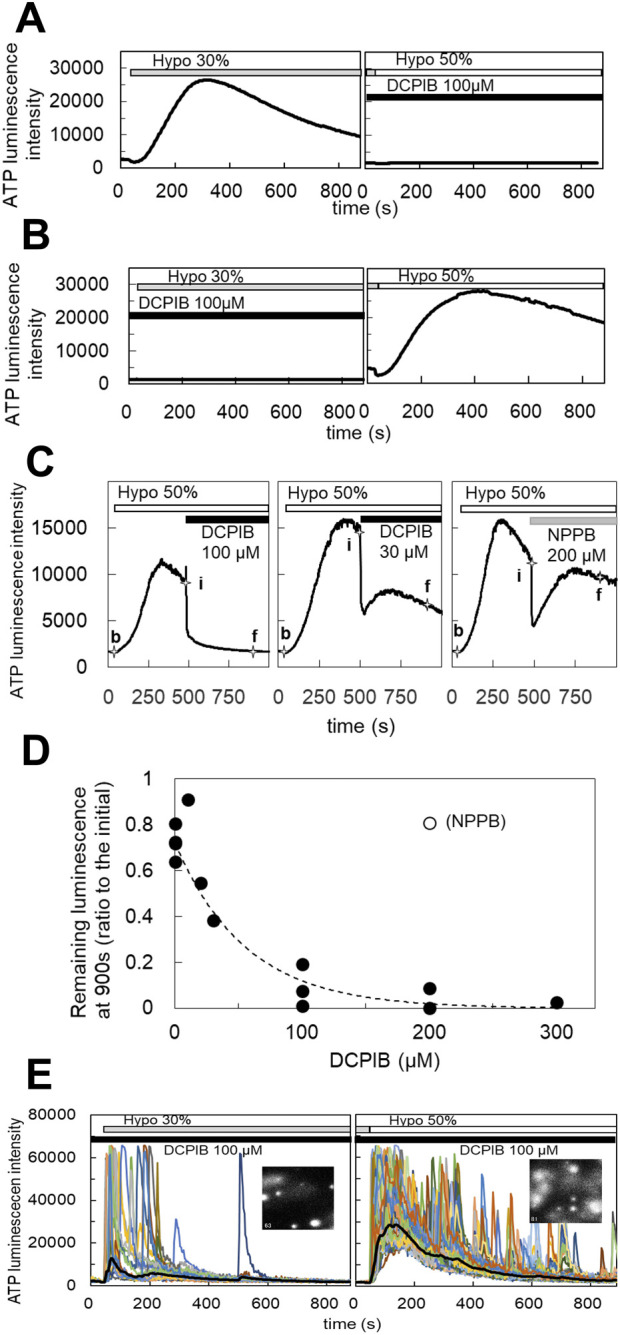
DCPIB, a blocker of the volume-regulated anion channels (VRACs), inhibited the slow diffuse response but not the sharp intermittent release. **(A)** Hypotonic stress (30%) induced the diffuse ATP release pattern in MCF10A cells. DCPIB treatment (100 µM) suppressed the response to subsequent 50% hypotonic stress. **(B)** DCPIB treatment (100 µM) suppressed the response to 30% hypotonic stress in MCF10A cells. After washout of DCPIB by several medium changes over 15 min, the ATP response recovered with subsequent 50% hypotonic stress. **(C)** The dose dependence of DCPIB in blocking diffuse ATP release were measured according to the level of decay of the diffuse ATP response after treatment with each concentration of DCPIB in MCF10A cells. The diffuse ATP release induced by 50% hypotonic stimulation (at 30 s; baseline intensity: b) was blocked by the perfusion of each concentration of DCPIB (at 480 s; initial intensity: i). The levels of decay were measured at 900 s (final intensity: f). Left trace: DCPIB 100 μM. center: DCPIB 30 μM. right: NPPB 200 µM. The blocking effect of DCPIB at each concentration was calculated based on the value of (f-b)/(i-b). **(D)** The dose dependence was determined based on the calculated value (f-b)/(i-b) at each concentration of DCPIB. The IC_50_ obtained by an exponential fitting curve was 38.5 µM. NPPB showed almost no effect, even at 200 µM. **(E)** The transient-sharp response in cholera toxin (100 ng/mL)-treated cells (MCF10A) was not affected by DCPIB treatment (100 µM) (see also Supplementary Movie S5). Other breast cell lines (MCF7, MDA-MB231) showed similar results.

We assessed the following inhibitors of ATP release: CBX and 10PANX for hemi channels, NPPB and DCPIB for Cl^−^ channels, CFTR (inh)-172 for CFTR, and a cocktail of brefeldin A, monensin and NEM, and clodronate for exocytosis. Among them, only DCPIB, a specific inhibitor of VRACs (Decher et al., 2001; Friard et al., 2017), effectively inhibited the diffuse pattern. As shown in Figures 2A,B, DCPIB treatment (100 µM) completely suppressed the diffuse ATP release pattern. The effects of DCPIB could be washed out (Figure 2B). The dose-dependent effects of DCPIB on the blockade of ATP release were measured according to the level of decay of the diffuse ATP response after treatment with each concentration of DCPIB (Figure 2C). DCPIB blocked the release of ATP in a dose-dependent manner, whereas NPPB (200 µM) did not affect the release of ATP (Figure 2C). The dose-response curve (Figure 2D) shows that the IC_50_ was 38.5 µM.

On the other hand, DCPIB did not block the transient-sharp release pattern in cholera toxin-treated cells (Figure 2E; Supplementary Movie S5) or primary cultured cells. On the contrary, DCPIB treatment sometimes enhanced or induced a transient-sharp release of ATP. Any of inhibitors mentioned above have no effect.

### 3.3 S1P induced the diffuse release of ATP which was blocked by DCPIB

**FIGURE 3:**
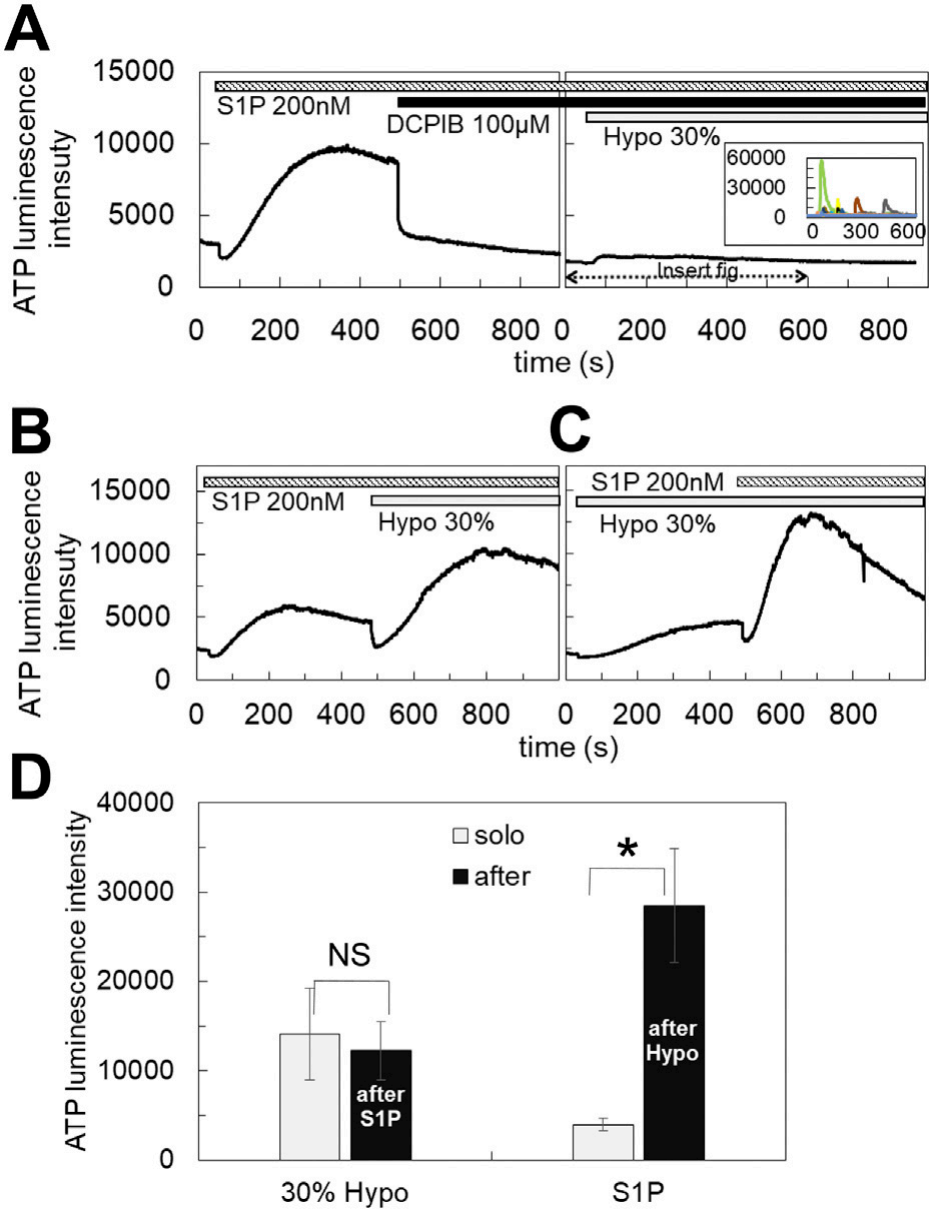
S1P induced the diffuse ATP release, which was blocked by DCPIB. **(A)** The application of S1P (200 nM) induced the diffuse ATP release pattern in MCF7 cells and DCPIB suppressed the response. The small response induced by 30% hypotonic stimulation after DCPIB treatment was due to the average of several intermittent sharp responses, as shown in the insert figure, which were occasionally induced by hypotonic stress in DCPIB-treated cells. **(B, C)** The response to S1P was additive to the effect of 30% hypotonic stimulation. Especially the response to S1P after 30% hypotonic stress was significantly enhanced in comparison to the sole administration of S1P **(D)**. Mean ± S.E. (N = 7), *t*-test; NS: no significance, *: *p* < 0.01.

The blockade of the diffuse ATP response by DCPIB suggests that VRACs contribute to the diffuse release of ATP. VRACs are activated not only by hypotonic stress but also isovolumetrically by various intracellular factors. The inflammatory mediator sphingosine-1-phosphate (S1P) has been reported to activate VRACs (Burow et al., 2015). The application of S1P (100 nM–1 µM) induced the release of ATP in MCF7 cells, exhibiting a slowly rising and diffuse pattern. This response had a comparable amplitude to that observed with 30% hypotonic stimulation and was blocked by DCPIB (Figure 3A), indicating that VRAC activation by S1P is involved in the diffuse ATP response. The responses to S1P (200 nM) and hypotonic stress (30%) were not competitive; rather, they were additive (Figures 3B,C). Furthermore, the response to S1P following hypotonic stimulation was enhanced compared to the response elicited by S1P alone (Figure 3D).

### 3.4 TGFβ enhanced the diffuse ATP release pattern induced by both hypotonic stress and S1P

**FIGURE 4:**
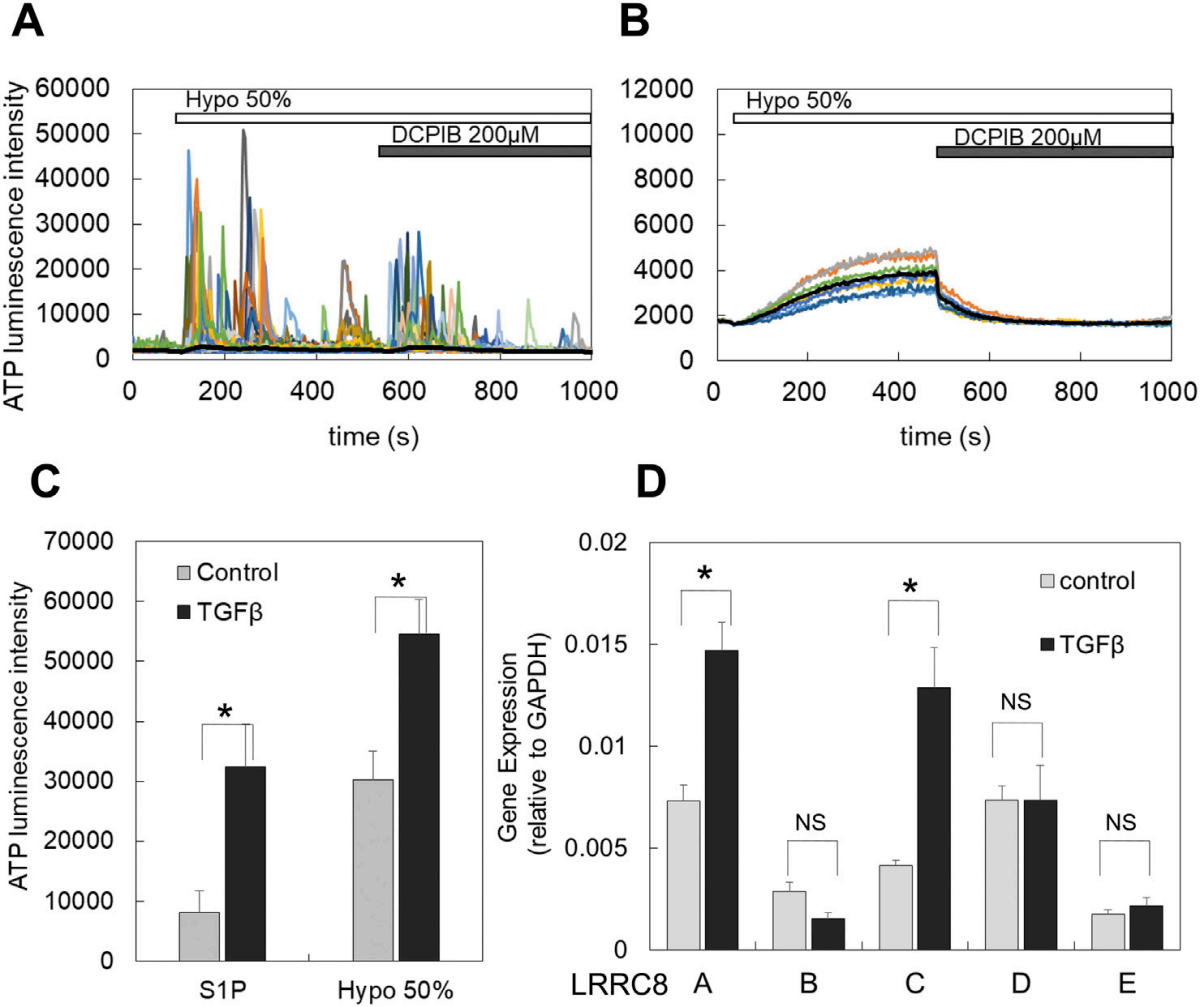
TGFβ treatment induced or enhanced the diffuse ATP release pattern and increased the expression of LRRC8 isoforms. **(A)** In primary cultured cells, the transient-sharp ATP release was typically induced by hypotonic stimulation (50%), and DCPIB did not block this release; rather, it enhanced the transient sharp ATP release. **(B)** After TGFβ treatment (10 ng/mL for 2 days) of sister cultured cells obtained from the same plating as A, the ATP release by hypotonic stress changed to the diffuse pattern, and DCPIB effectively blocked this ATP release. **(C)** In breast cell lines, TGFβ enhanced the diffuse ATP release induced by both S1P and hypotonic stress. The peak intensity of ATP luminescence induced by S1P and hypotonic solutions was measured in each cell line, comparing control and TGFβ-treated cells (as shown in Supplementary Figure S2A). A plot of the average of all data from 3 cell lines indicates that TGFβ treatment significantly enhanced the diffuse release of ATP by both S1P and hypotonic stress. Mean ± S.E. (N = 12), *t*-test; *: *p* < 0.001. **(D)** The LRRC8 isoforms **,**(A, B, C, D, E) expressed in breast cell lines and TGFβ treatment enhanced the expression. The levels of these isoforms in both control and TGFβ-treated cells were measured using RT-qPCR and normalized to GAPDH for each sample. All data from 3 cell lines were averaged. Differences among the cell lines are presented in Supplementary Figure S2B. Mean ± S.E. (N = 9), *t*-test; NS: no significance, *: *p* < 0.01.

Transforming growth factor β (TGFβ) plays a critical role in mammary development and carcinogenesis by regulating various cellular activities, including the epithelial-mesenchymal transition (EMT) (Moses and Barcellos-Hoff, 2011). In primary cultured mammary epithelial cells, hypotonic stimulation induced the transient-sharp ATP release pattern (Figures 1B, 4A). In contrast, treatment with TGFβ (10 ng/mL for 1–3 days) altered the ATP release pattern to a diffuse one (Figure 4B). In breast cell lines, the peak intensity of ATP luminescence induced by S1P (1 μM) followed by the application of a hypotonic solution (50%) was measured (Supplementary Figure S2A). TGFβ treatment enhanced the diffuse ATP release induced by both S1P and the hypotonic solution (Figure 4C; summarized data for all tested breast cell lines, and Supplementary Figure S2B; for each individual breast cell line).

### 3.5 LRRC8A isoforms were expressed in breast cell lines, and TGFβ enhanced the expression

The molecular entity of VRACs has been identified as LRRC8, which exists in five isoforms (A to E) (Voss et al., 2014; Qiu et al., 2014) that form a hexameric heteromer. However, there are still points of contention regarding the specific contributions of each isoform to the channel functions of VRACs (Jentsch et al., 2016). We investigated the expression of LRRC8 isoforms (A, B, C, D, and E) in breast cell lines using RT-qPCR. In control cultures, all isoforms were expressed (Figure 4D). Among these, LRRC8A, C, and D were prominently expressed, while LRRC8B and E showed minimal expression. Following TGFβ treatment, the expression levels of LRRC8A and C were further enhanced (Figure 4D). The extent of this enhancement varied somewhat among the different cell lines, as illustrated in Supplementary Figure S2C.

### 3.6 Gene silencing with shRNA for LRRC8A

**FIGURE 5:**
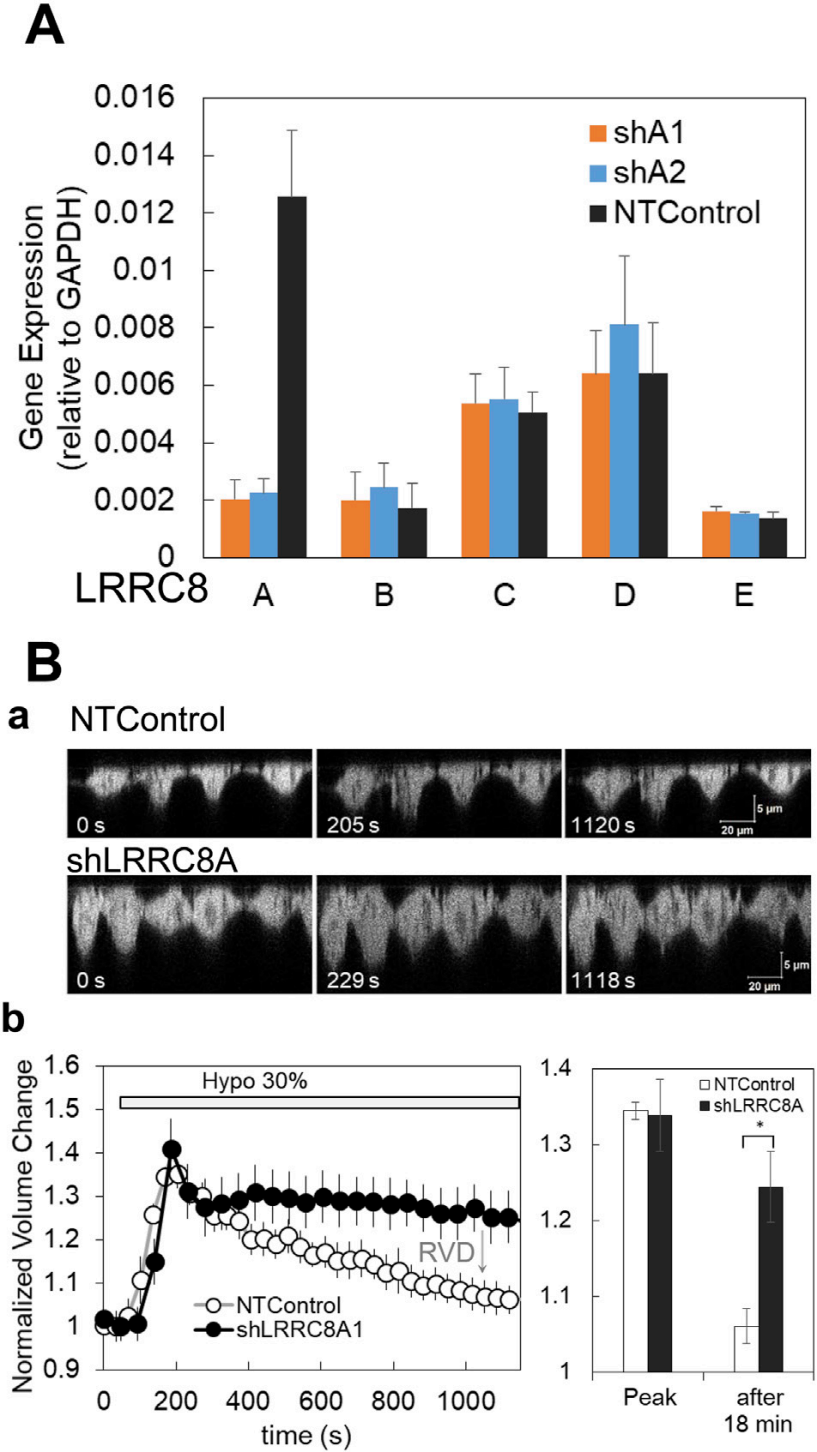
Gene silencing with shRNA for LRRC8A and suppression of the regulatory volume decrease (RVD) in knockdown cells. **(A)** Two types of shRNA for LRRC8A (shA1 and shA2) suppressed the LRRC8A gene expression but did not affect the expression of other LRRC8 isoforms (LRRC8B to E) in breast cell lines. Average of the data from 3 cell lines is shown. Differences among the cell lines are shown in Supplementary Figure S3. The expression of LRRC8 isoforms, (A, B, C, D, E) in the cells treated with non-targeting control (NTControl), shA1 and shA2 were measured by RT-qPCR and normalized to the GAPDH within each sample. **(B)** The knockdown of LRRC8A (shA1) suppressed the regulatory volume decrease (RVD) after the induction of volume increase by hypotonic stress. (a), A time series of X-Z cross-section of MDA-MB231 cells, which expressed GFP in cytosol, was monitored with a confocal microscope. Upper: NTControl, lower: shLRRC8A treated cells. (b), The time course of the cell volume changes induced by hypotonic stress, which was calculated as the reciprocal of the fluorescence intensity of a specific unit area of the cytosol and normalized to the initial value. Control cells (open circle) showed a volume increase following a volume decrease after hypotonic stress, which means RVD. RVD was suppressed in LRRC8A knock-down cells (closed circle). The relative volume changes at the peak and 18 min after the application of the hypotonic solution in both cell types were plotted. Values represent the mean ± S.E. (N = 7), *t*-test; *: *p* < 0.001.

To confirm the contribution of VRACs to the ATP release pathway associated with the diffuse pattern, we knocked down LRRC8A, an indispensable subunit of VRACs, using shRNA. LRRC8A silencing was achieved through retrovirus-mediated gene transfer. Two shLRRC8A vectors (shA1 and shA2) and a non-targeting control vector (NTControl) were cloned in three breast cell lines. RT-qPCR analysis of the LRRC8 isoforms (A to E) demonstrated that both shLRRC8A1 and shLRRC8A2 vectors effectively suppressed the expression of LRRC8A without affecting the other isoforms (B, C, D, and E) across the average of the 3 cell lines (Figure 5A) and in each individual cell line: MDA-MB231, MCF7, and MCF10A (Supplementary Figure S3).

We first checked whether the deletion of LRRC8A affected a critical VRACs function in the cells—regulatory volume decrease (RVD)—following an increase in volume by hypotonic stress. Hypotonic stress (30%) resulted in an increase in cell height, as observed in the perpendicular cross-sectional images obtained through a confocal X-Z-T scan in both shLRRC8A1 and control cells at approximately 200 s. Subsequently, the cell height decreased in control cells; however, it remained unchanged in shLRRC8A1 cells at around 1,000 s (Figure 5Ba). The normalized cell volume change, calculated as the reciprocal of fluorescence intensity of a specific unit area of the cytosol and normalized to a value obtained just prior to stimulation, increased rapidly until 200 s in both control and shLRRC8A1 cells. After this point, the cell volume gradually decreased in control cells, indicating normal RVD, while it remained constant in shLRRC8A1 cells (Figure 5Bb, left). The normalized volume changes at the peak and 18 min after hypotonic stimulation in both cell types were plotted (Figure 5Bb, right). These results demonstrate that the deletion of LRRC8A impairs the RVD function in the cells.

### 3.7 LRRC8A knockdown suppressed the diffuse release of ATP induced by both hypotonic stress and S1P

**FIGURE 6:**
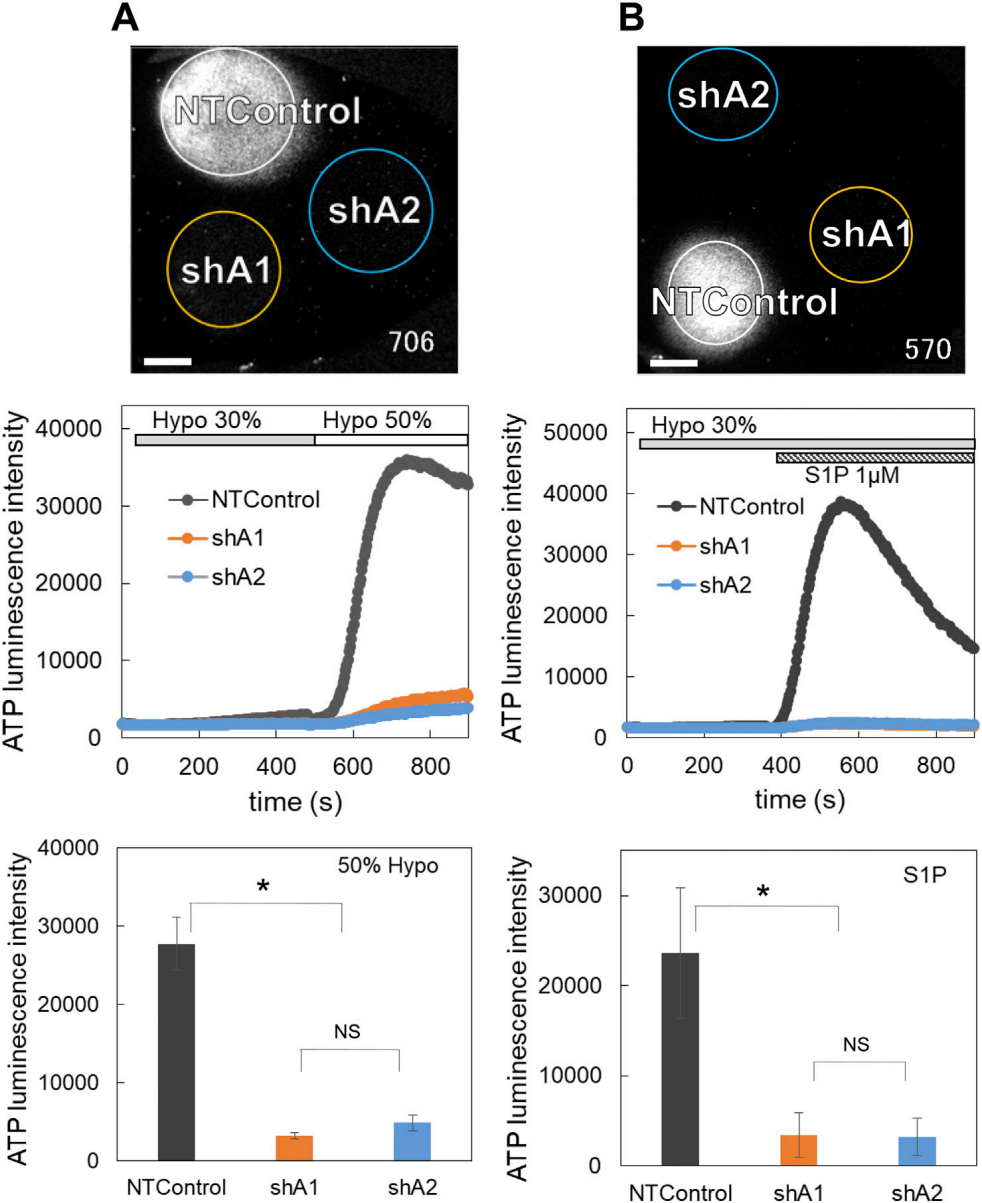
The knockdown of LRRC8A suppressed the diffuse ATP release induced by both hypotonic stress **(A)** and S1P **(B)**. **(A)** ATP release induced by hypotonic stress (30% and 50%, applied sequentially) was measured in the cells transfected with two shLRRC8A vectors (shA1 and shA2) and NTControl simultaneously, which were cultured on a cover glass (upper image and Supplementary Movie S6). Scale is 2 mm. Middle trace is the time course of measured luminescence intensity. An example of MCF10A cells. Other cell lines (MCF7, MDA-MB231) showed similar results. Lower graph shows suppression effects of shA1 and shA2 on the ATP release induced by hypotonic stress (50%). Summary of all data from every cell lines. Mean ± S.E. (N = 15), *t*-test; *: *p* < 0.001. **(B)** ATP release induced by S1P (1 μM) subsequent to 30% hypotonic stress was measured in the cells transfected with two shLRRC8A vectors (shA1 and shA2) and NTControl simultaneously, which were cultured on a cover glass (upper image and Supplementary Movie S7). Scale is 2 mm. An example of MCF7 cells. Middle trace is the time course of measured luminescence intensity. Other breast cell lines (MCF10A, MDA-MB231) showed similar results. Lower graph shows suppression effects of shA1 and shA2 on the ATP release induced by S1P. Summary of all data from every cell lines. Mean ± S.E. (N = 6), *t*-test; *: *p* < 0.01.

The ATP release induced by hypotonic stress was measured in cells transfected with two shLRRC8A vectors (shA1 and shA2) and NTControl. Three types of cells (shA1, shA2, and NTControl) were cultured on a cover glass, and the changes in ATP luminescence induced by 30% and 50% hypotonic solutions in each cell line were measured simultaneously. The application of hypotonic solution elicited a significant release of ATP in NTControl cells, but not in shA1 or shA2 cells (MCF10A, Figure 6A, upper figure and middle traces; Supplementary Movie S6). The lower graph in Figure 6A summarizes all data on the inhibitory effects of LRRC8A deletion on the peak intensity of ATP release by hypotonic stress (50%) in 3 cell lines. The shA1 and shA2 vectors reduced ATP release by 88% and 82%, respectively.

The deletion of LRRC8A by both shRNAs also inhibited the S1P-induced release of ATP (MCF7, Figure 6B, upper figure and middle traces; Supplementary Movie S7). The lower graph in Figure 6B summarizes all data on the inhibitory effects of shA1 and shA2 on the S1P-induced ATP release in 3 cell lines. Both shA1 and shA2 reduced ATP release by 86%.

### 3.8 LRRC8A knockdown suppressed the cancer progression in nude mouse xenograft model

**FIGURE 7:**
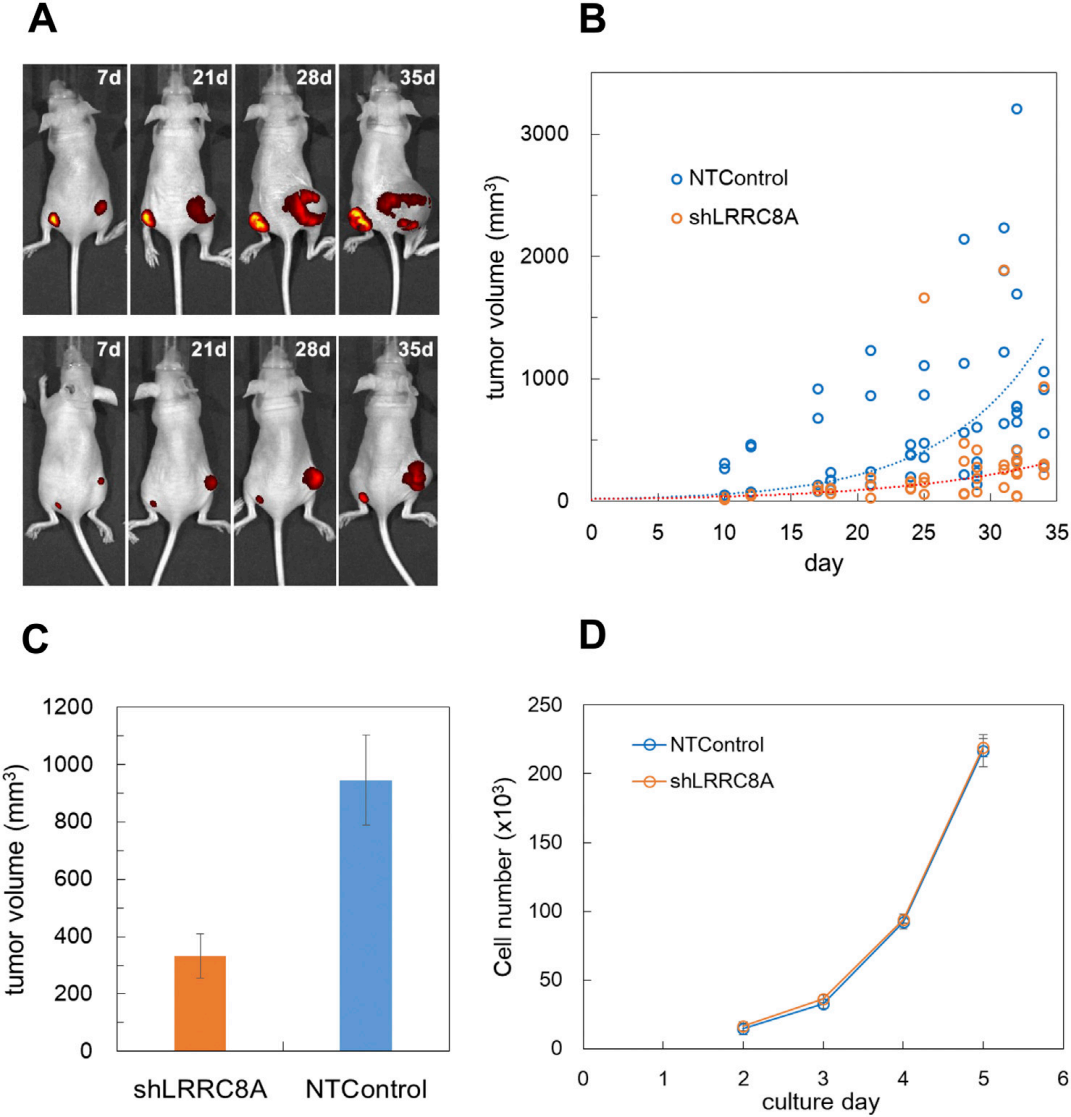
LRRC8A knockdown suppressed the cancer progression in nude mouse xenograft model. **(A)** GFP-expressing MDA-MB231 cells were subcutaneously injected into the lower back of nude mice, and tumor progression was monitored using fluorescence with an *in vivo* imaging system (IVIS Spectrum). LRRC8A knockdown cells (shA1) were injected on the left side, while NTControl cells were injected on the right side. A total of 1 × 10^6^ cells (1 × 10^7^ cells/mL, 100 µL) were injected for each condition. Two representative mice (out of 16 total) exhibiting tumor growth over time are shown. **(B)** The tumor size, measured transdermally using a digital caliper, was plotted against the day following injection. Tumor volume was estimated as an ellipsoid using equation L*W^2^*4pai/3/8 (where L: length, W: width). This data encompasses all tested mice (4 experiments, 4 mice per experiment, over a duration of 7–35 days). The broken lines indicate the curves of exponential fitting for both cases, with τ values of 7.65 for the control and 11.4 for the knockdown cells. **(C)** The average tumor sizes at days 28–34 were compared. The tumor volume with LRRC8A knockdown cells was significantly smaller than that with NTControl cells (n = 24, *p* = 0.0011, *t*-test). **(D)** The growth rate of each cell in culture did not differ between LRRC8A knockdown cells and NTControl cells. The number of cells in each culture dish was counted daily using a hemocytometer following dissociation. N = 3.

To evaluate the *in vivo* impact of ATP release via VRACs on tumor progression, LRRC8A knockdown (shA1) and control MDA-MB231 cells were subcutaneously injected into the left and right sides of the lower back of nude mice, respectively. Both cell lines expressed GFP in the cytosol, allowing for tumor progression to be monitored through fluorescence using an *in vivo* imaging system. After 1 week, a small nodule was observed, which grew significantly larger over the following weeks (Figure 7A). This figure illustrates a slower tumor growth rate on the left side compared to the right side. Tumor size was also measured transdermally using a caliper. Tumor volume was estimated from the length and width and was plotted against days after injection (Figure 7B). In Figure 7B, LRRC8A knockdown cells exhibited a slower tumor growth rate compared to NTControl cells. The sizes of tumors grown on days 28–34 were significantly smaller in LRRC8A knockdown cells than in NTControl cells (Figure 7C). This difference was not caused by differences in the growth rates of either cell type because there was no significant difference in the growth rate in subculture between LRRC8A knockdown and NTControl cells (Figure 7D).

## 4 Discussion

Our real time ATP imaging study uncovered two unique patterns of ATP release in mammalian epithelial cells under hypotonic stress. One is a diffuse-slow but substantial and prolonged pattern of ATP release in breast cell lines and the other is intermittently released with transient-sharp peaks in differentiated cells such as primary culture. The diffuse ATP release pattern was blocked by DCPIB and suppressed by LRRC8A knockdown using shRNA. Thus, it was concluded that VRACs contributed to the diffuse release of ATP by hypotonic stress in breast cell lines. On the contrary, the transient-sharp pattern was not suppressed by DCPIB (Figures 2E, 4A) nor any other blockers. LRRC8A-knockdown also did not affect. Thus, the pathway of transient-sharp ATP release remains unclear at present. However, transient-sharp ATP release is so prominent in primary cultured or differentiated cells that it may contribute to certain cell functions, which remains to be elucidated.

VRACs facilitate the passage of various substances, including ATP (Hisadome et al., 2002; Burow et al., 2015; Gaitán-Peñas et al., 2016), glycine, aspartate, glutamate, GABA, taurine, myo-inositol, and lactate, in addition to Cl^−^ (Lutter et al., 2017; Schober et al., 2017). It was debatable whether or not VRACs are a pathway of ATP release (Sabirov and Okada, 2005; Liu et al., 2009). However, it is now clear that variations in the heteromeric subunit structures of the LRRC8 hexamer account for these variations in the properties of VRACs (Planells-Cases et al., 2015; Gaitán-Peñas et al., 2016; Jentsch et al., 2016; Okada et al., 2017). In addition to the indispensable component LRRC8A, the expression of LRRC8D enhances large solute permeability (Planells-Cases et al., 2015; Lutter et al., 2017; Schober et al., 2017), while LRRC8C affects the permeability of charged osmolytes (Schober et al., 2017); these are supported by cryo-EM structure studies (Deneka et al., 2018; Nakamura et al., 2020). According to a recent study by Lahey et al. (2020), LRRC8C facilitates the transport of cGAMP (2′3′-cyclic-GMP-AMP), while LRRC8D impedes it. There remains ambiguity concerning the role of each subunit and its combinations. Our results suggest that in addition to LRRC8A, LRRC8C and D are important subunits for the release of ATP, given their markedly high expression and TGFβ-dependent increases in breast cell lines (Figure 4D; Supplementary Figure S2C).

VRACs are not only a key element of vertebrate cell volume regulation but also participates in various physiological and pathophysiological processes (Okada et al., 2009; Pedersen et al., 2016). In human cervical cancer cells, the inhibition of VRACs resulted in G0/G1 arrest (Shen et al., 2000). The VRAC activity in cancerous nasopharyngeal epithelial cells was significantly greater than that in normal human nasopharyngeal epithelial cells, which is consistent with their enhanced growth capacity (Zhu et al., 2012). The expression of LRRC8A is elevated in the tissues of colorectal cancer patients, which correlates with a shortened survival time, and the knockdown of LRRC8A in colon cancer cells inhibits tumorigenesis in a xenograft model (Zhang et al., 2018; Zhang et al., 2024). In other various human cancers, VRACs have been reported to contribute to tumor progression, including hepatocellular (Lu et al., 2019), gastric (Kurashima et al., 2021), pancreatic (Xu et al., 2022), cervical (Chen et al., 2023), renal, cutaneous, glioma, melanoma, and sarcoma (Carpanese et al., 2024). All of these cancers are associated with a worse prognosis for patients. The mechanisms underlying tumor progression involving VRACs are complex and not yet fully elucidated. They encompass multiple pathways both within cancer cells and in the surrounding tumor microenvironment. In cancer cells, the enhancement of proliferation, migration, metastasis, and infiltration, along with the suppression of apoptosis, occurs through various intracellular signaling processes, including MAPK, PIP, p53, PKC, m5C RNA modification, and the reconstruction of the cytoskeleton. These processes are influenced by the stress response of volume-regulated anion channels (VRACs). In contrast, another study (Liu and Stauber, 2019) found that LRRC8A knockdown or gene knockout did not affect cell proliferation and migration in several cultured cell types, including myoblasts, colon cancer cells, and glioblastoma cells. In the tumor microenvironment, VRACs facilitate the release of small molecules, such as cGAMP (Lahey et al., 2020) and ATP (Burow et al., 2015; this paper), which modify the environmental conditions. cGAMP, a paracrine innate immune messenger, is imported into cells to activate the stimulator of interferon genes (STING), thereby enhancing antitumor immunity (Wang et al., 2024). The effects of ATP on tumor progression vary depending on the receptors and cell types involved (Zhou et al., 2015; Vultaggio-Poma et al., 2020; Alvarez et al., 2022). Additionally, adenosine, a byproduct of ATP hydrolysis, serves multiple functions, primarily suppressing immune attacks on tumor cells through the activation of specific immune cells. Among these various pathways, the specific mechanisms of action *in vivo* within cancer tissues differ significantly based on the type of cancer, its state, and the tumor microenvironment. Consequently, VRACs are implicated in the development and progression of cancer and represent a potential target for cancer therapeutics (Xu et al., 2020).

In our study utilizing the nude mouse xenograft model, the progression of tumors formed by VRAC knockdown breast cancer cells was significantly slower than that of control cells, despite no notable difference in the growth rate during subculture between the 2 cell types (Figure 7). Thus the difference is likely attributed to variations in the tumor microenvironment created by the implanted cells. Our findings suggest that the absence of VRACs markedly inhibits tumor growth due to a resultant limitation in cellular ATP supply. Adenosine suppresses the immune response against cancer by inhibiting natural killer (NK) cells and activating regulatory T cells through the A2A adenosine receptor (Antonioli et al., 2013a). Xenograft experiments are conducted using nude mice because they lack a thymus-derived immune system. However, an alternative immune system persists (Hanna, 1980; Panaampon et al., 2021), which includes NK cells and peripherally derived regulatory T cells. The removal of VRACs in cancer appears to counteract the suppression of anti-tumor immune responses caused by chronically elevated adenosine. To substantiate this, we are now planning *in vivo* ATP imaging and the identification of immune cells infiltrating the tumor site.

VRACs were isovolumetrically activated by intracellular GTPγS, purinergic signaling, bradykinin, mGluR, ROS and Ca^2+^ signaling (Pedersen et al., 2016; Okada et al., 2019; Bertelli et al., 2021), as well as by a decrease in ionic strength (Syeda et al., 2016). S1P also induced the diffuse release of ATP isovolumetrically, and this was blocked by DCPIB and suppressed in LRRC8A-knockdown cells (Figures 3, 6B; Furuya et al., 2021a). This shows that S1P activated VRACs and induced the release of ATP. Interestingly, S1P induced release of ATP enhanced after hypotonic stress (Figure 3D), implying that S1P receptors on the membrane folding, such as caveolae, might appear during the cell expansion by the hypotonic stress. We suggested the involvement of its G protein-coupled receptors S1PR1 and R2 to activate VRACs (Furuya et al., 2021a). Recently, VRAC activation by S1P is reported to be mediated by S1PR1 coupled to Gi family (Kostritskaia et al., 2024). S1P induced ATP release via VRACs formed an autocrine link between inflammatory sphingolipid and purinergic signaling in macrophages (Burow et al., 2015) and microglia (Zahiri et al., 2021; Chu et al., 2023). S1P is an inflammatory mediator and is produced by sphingosine kinase, which is activated by several inflammatory signaling molecules, including bacterial lipopolysaccharide (LPS), PDGF, TNFα, thrombin, IgE-bound antigen and ATP (Burow et al., 2015; Burow and Markwardt, 2014). S1P is rich in the cancer microenvironment (Nagahashi et al., 2012) and plays important roles in cancer progression via diverse pathways of its G-protein coupled receptors, which implicates S1P pathway as a therapeutic target (Pyne and Pyne, 2010; Ogretmen, 2018; Nagahashi et al., 2018). It is plausible that the induction of the release of ATP via VRACs is an important function of S1P in the cancer microenvironment (Furuya et al., 2021a).

In addition to the diffuse ATP release pattern induced via VRACs, the transient-sharp ATP release pattern was induced by hypotonic stress in mammary epithelial cells (Figures 1B,D). The transient-sharp pattern usually appeared in primary cultured cells, and the diffuse ATP release pattern was observed in breast cell lines, although both patterns co-existed to varying degrees in the cells. Sometimes spontaneous release of ATP with the transient-sharp pattern was observed without any stimulation. Cholera toxin treatment in breast cell lines changed the ATP release pattern from diffuse to transient-sharp (Figure 1D). Cholera toxin is produced by *Vibrio cholerae* and is a multifunctional protein that influences various cells, including the immune system, and acts as an anti-inflammatory agent (Bharati and Ganguly, 2011; Baldauf et al., 2015). Cholera toxin has been shown to suppress carcinogenesis in colon cancer (Doulberis et al., 2015) and may exert contact inhibition by binding to glycosphingolipids in MCF10A cells (Huang et al., 2017). A function of cholera toxin is to activate adenylate cyclase, which increases the intracellular cAMP level. Cholera toxin also exerts its activity through the nontoxic cholera toxin B subunit, which specifically binds to the surface receptor GM1 ganglioside on lipid rafts (Baldauf et al., 2015; Day and Kenworthy, 2015) and works as a raft cross-linker or triggers endocytosis of the binding area. Our preliminary finding that the cholera toxin B subunit mimicked this effect, but dibutyryl-cAMP treatment did not induce the effect suggested the latter case. Interestingly, GM1 is also a sphingosine kinase activator that induces the production of S1P (Wang et al., 1996).

In contrast to the effect of cholera toxin, TGFβ treatment converted the transient-sharp ATP release to the diffuse pattern in primary cultured mammary epithelial cells (Figures 4A,B) and enhanced the diffuse pattern in breast cell lines (Figure 4C). TGFβ, which was first implicated in mammary epithelial development, is critically important for mammary morphogenesis and secretory function (Moses and Barcellos-Hoff, 2011; Daniel et al., 1989). TGFβ is also known to exist abundantly at tumor sites and plays central roles in carcinogenesis (Syed, 2016). TGFβ signaling helps to regulate crucial cellular activities, such as cell growth, differentiation, apoptosis, motility, invasion, extracellular matrix production, angiogenesis, and immune response. However, the role and signaling pathway of TGFβ are completely cell-context dependent. TGFβ is an important inducer of EMT in both development and carcinogenesis (Syed, 2016; Martínez-Ramírez et al., 2017). There have been some reports of TGFβ inducing the EMT in mammary epithelial cell lines, including MCF10A (Zhang et al., 2014), MDA-MB231 and MCF7 (Romagnoli et al., 2012; Chen et al., 2017). Although the mechanism by which TGFβ influences the release of ATP via VRACs is unclear, EMT may be associated with changes in the pattern of ATP release.

In the present study, we used three breast cell lines that originated from different tissue states. MDA-MB231 cells were derived from adenocarcinoma and are highly aggressive with triple-negative properties (Cailleau et al., 1974). MCF7 cells were a ductal carcinoma cell line (Soule et al., 1973). MCF10A cells originated from benign tumors of fibrocystic disease and are non-carcinogenic, although not normal karyotypically (Soule et al., 1990). These cell lines possess different characteristics, as demonstrated by gene and protein expression profiling (Charafe-Jauffret et al., 2006). However, all of these cell lines are immortal and exhibit undifferentiated properties under usual culture conditions.

Treatment with cholera toxin, which suppresses inflammation and carcinogenesis and occasionally induces differentiation in various types of cells, suppressed the diffuse ATP release pattern and induced the transient-sharp release pattern. Treatment with TGFβ, which sometimes induces carcinogenesis and EMT, induces the diffuse ATP release pattern in primary cultured cells and enhances the diffuse ATP release pattern in breast cell lines. In addition to mammary cell lines, a lung cancer cell line (A549) exhibited a similar diffuse pattern (Furuya et al., 2014). The diffuse ATP release pattern was rarely observed in primary cultured cells of mammary glands and subepithelial fibroblasts in the intestine. These results suggest that the appearance of diffuse ATP release depends on the undifferentiated state of the cells, including cancer cells. The slow, diffuse, yet substantial and prolonged release of ATP via VRACs is demonstrated as a source of ATP—and accordingly adenosine—in the cancer microenvironment. This process certainly plays a significant role in the functions of these molecules during cancer progression. These VRACs’ unique features make them promising candidates for cancer therapy in the microenvironment.

## Data Availability

The original contributions presented in the study are included in the article/Supplementary Material, further inquiries can be directed to the corresponding author.
