# Supplementary material for: Volume-regulated anion channels conduct ATP in undifferentiated mammary cells and promote tumorigenesis in xenograft nude mouse
Source: Front Cell Dev Biol. 2025 Jan 15;12:1519642. doi: 10.3389/fcell.2024.1519642 (PMC11774906; doi:10.3389/fcell.2024.1519642)
Supplement: Supplementary file 2 [file DataSheet1.pdf]

Suppl. Fig. S1

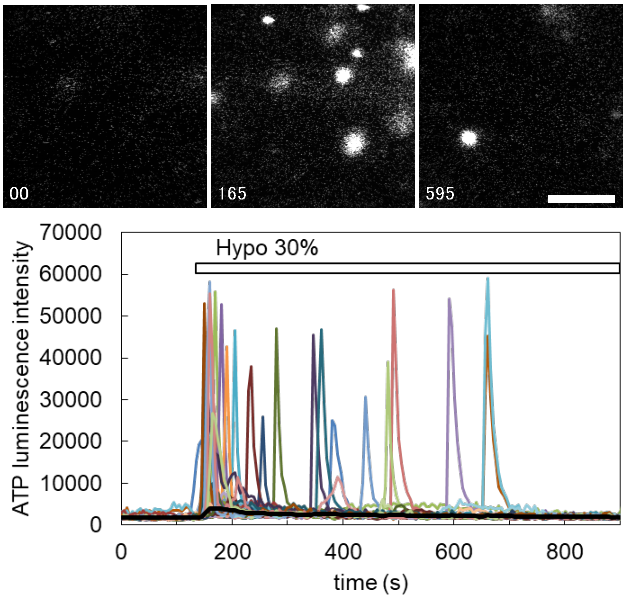

Fig. S1. Primary culture of subepithelial fibroblasts in rat intestinal villi released ATP with transient-sharp pattern in response to 30% hypotonic stress. Subepithelial fibroblasts were isolated and cultured from rat intestinal villi by the method described in Furuya S & Furuya K (2007), and treated with endothelin 1 100 nM, which made the cells differentiate as myofibloblasts. Scale bar represents 1 mm.

Suppl. Fig. S2

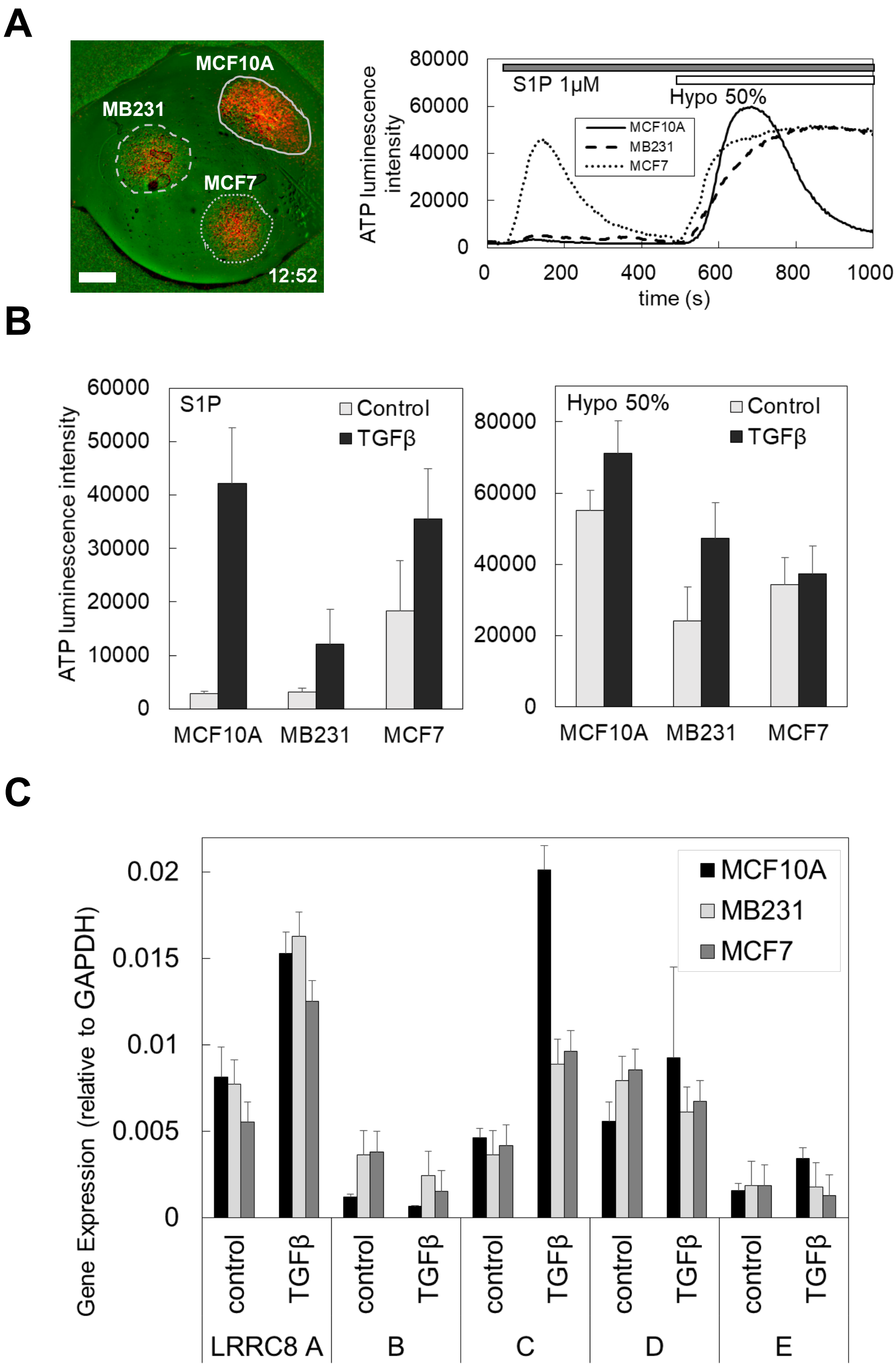

Fig. S2.
A: Three types of breast cell lines (MCF10A, MDA-MB231, MCF7) cultured on 3 collagen-gel patches each on a 22-mm ϕ cover glass placed in a small perfusion chamber. Left image shows an example of the measurement of the ATP release using this system. Scale bar is 2 mm. In right trace, the time courses of the typical ATP response induced by S1P (1 μM) and subsequently applied hypotonic solution (50%) are shown. B: TGF β treatment enhanced the diffuse release of ATP induced by both S1P and hypotonic stress in each breast cell line. C: TGF β treatment enhanced the expression of LRRC8 isoforms in each breast cell line. Increases in LRRC8A and C were prominent.

Suppl. Fig. S3

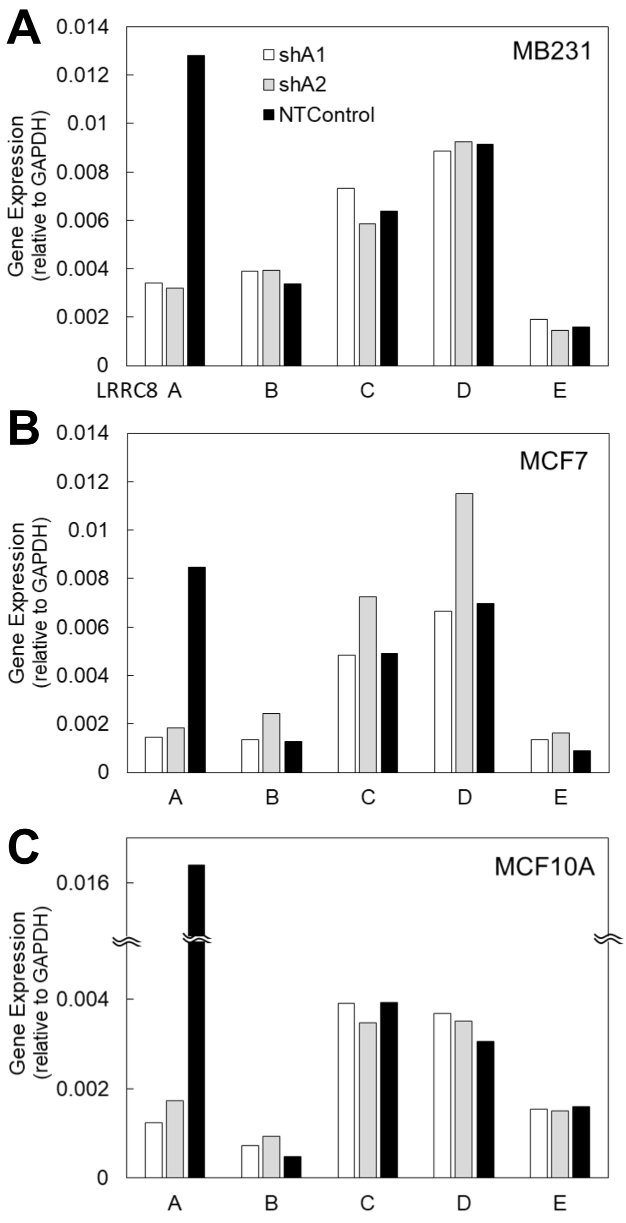

Fig. S3. Suppression of gene expression of LRRC8A by 2 types of shRNA (shA1 and shA2) in each breast cell line. A: MDA-MB231, B: MCF7, C: MCF10A.

Supplemental Table S1. RT-qPCR primers and shRNA target sequences

RT-qPCR primers

Target		Sequence(5'-3')	Oligo name (TaKaRa)
LRRC8A	F	CGCAGATAGCAGAGCCATCC	HA148182–F
LRRC8A	R	GCAATCATCAGCATGACGATAGAGA	HA148182–R
LRRC8B	F	GCTATAACCACTTGACCTTCATTCC	HA272096–F
LRRC8B	R	TTGCACTGAAACAGCCCATC	HA272096–R
LRRC8C	F	CATGATCGGCGTGTTTGGA	HA289034–F
LRRC8C	R	AGTGGTACTGGCAACTGCTTGAGA	HA289034–R
LRRC8D	F	AGAGAGCGCCCTGAGAGAACAG	HA164457–F
LRRC8D	R	CATGGTTTCAGGATTCGGTAAGTTG	HA164457–R
LRRC8E	F	GCCTTGCTCCCATCCCTAGAA	HA249796–F
LRRC8E	R	ATATGTGGCACTAATTGAGCTGGAA	HA249796–R
GAPDH	F	GCACCGTCAAGGCTGAGAAC	HA067812–F
GAPDH	R	TGGTGAAGACGCCAGTGGA	HA067812–R

shRNA target sequences

LRRC8A	1	GAGCGCAGTATTTGGATAA	shA1
LRRC8A	2	GGCACGTTTTAGAGTCTCT	shA2
